# Bidirectional Temporal Association Between Cytomegalovirus Reactivation and Graft-Versus-Host Disease Following Allogeneic Hematopoietic Stem Cell Transplantation: A Single-Center Real-World Cohort Study

**DOI:** 10.3390/v18080874

**Published:** 2026-08-11

**Authors:** Emel İşleyen, Simten Dağdaş, Bircan Kayaaslan, Funda Ceran, Mehmet Sezgin Pepeler, Ayşe Kaya, Gülten Korkmaz, Merve Ecem Erdoğan Yön, Fahir Öztürk, Ahmet Ceylan, Derya Kayardı, Gülsüm Özet

**Affiliations:** 1 Department of Hematology, Ankara Bilkent City Hospital, Ankara 06800, Türkiye; simtendagdas@gmail.com (S.D.); ceranf@gmail.com (F.C.); dr.sezgin44@gmail.com (M.S.P.); drgulten@gmail.com (G.K.); merveecemerdogan@hotmail.com (M.E.E.Y.); drfahir@gmail.com (F.Ö.); ahmetceylanac@gmail.com (A.C.); gulsumozet@gmail.com (G.Ö.); 2 Department of Infectious Diseases and Clinical Microbiology, Ankara Bilkent City Hospital, Ankara 06800, Türkiye; drbican@gmail.com (B.K.); dr.aysekaya09@hotmail.com (A.K.); deryakyrdi@gmail.com (D.K.)

**Keywords:** cytomegalovirus, graft-versus-host disease, allogeneic hematopoietic stem cell transplantation, CMV reactivation, pre-emptive therapy, letermovir, survival

## Abstract

Background: Cytomegalovirus (CMV) reactivation and graft-versus-host disease (GVHD) are major causes of morbidity and non-relapse mortality following allogeneic hematopoietic stem cell transplantation (allo-HSCT). Although their association has been extensively investigated, the temporal relationship between these complications and their impact on survival remain incompletely understood, particularly in centers where routine letermovir prophylaxis is unavailable and CMV is managed using a pre-emptive treatment strategy. This study evaluated the bidirectional temporal association between CMV reactivation and GVHD using time-dependent analyses and assessed their impact on survival in a real-world allo-HSCT cohort. Methods: We retrospectively analyzed 100 consecutive adult patients who underwent allo-HSCT for hematologic malignancies between January 2016 and February 2025. CMV surveillance was performed by weekly quantitative CMV-DNA PCR, and reactivation was managed using a standardized pre-emptive treatment strategy. Patients who relapsed or died within the first 100 days after transplantation were excluded. The incidence, temporal sequence, risk factors, and prognostic impact of CMV reactivation and GVHD were evaluated. Overall survival (OS) and relapse-free survival (RFS) were estimated using the Kaplan–Meier method, while temporal associations were assessed using time-dependent Cox regression analyses. Results: CMV reactivation occurred in 72 patients (72%), whereas GVHD developed in 51 patients (51%). Among patients who experienced both complications, CMV reactivation preceded GVHD in 32 patients, while GVHD preceded CMV reactivation in 14 patients. Older recipient age (42.3 ± 13.5 vs. 35.4 ± 11.2 years, *p* = 0.018) and older donor age (37.2 ± 10.9 vs. 32.5 ± 9.7 years, *p* = 0.048) were associated with CMV reactivation. Older donor age was also associated with GVHD development (38.1 ± 10.7 vs. 33.6 ± 10.5 years, *p* = 0.037). The median time to CMV reactivation was 37 days, and CMV end-organ disease occurred in 13.9% of affected patients. No significant differences in OS or RFS were observed according to CMV reactivation or GVHD status in the day-100 landmark cohort. In the AML subgroup, both CMV reactivation and GVHD showed a trend toward inferior OS and RFS without reaching statistical significance. Time-dependent Cox regression demonstrated that CMV reactivation independently increased the subsequent risk of GVHD (HR 2.93, 95% CI 1.51–5.69; *p* = 0.001), whereas GVHD also independently increased the subsequent risk of CMV reactivation (HR 1.98, 95% CI 1.06–3.71; *p* = 0.032). Conclusions: CMV reactivation was highly prevalent after allo-HSCT and frequently preceded GVHD, supporting a bidirectional temporal relationship between these complications. Older donor age was associated with both CMV reactivation and GVHD. Although survival did not significantly differ according to CMV or GVHD status in this day-100 landmark cohort, clinically important CMV-related complications, including end-organ disease and platelet engraftment failure requiring eltrombopag, remained common. These findings indicate that a standardized pre-emptive strategy did not completely prevent CMV-associated morbidity. Because letermovir was not evaluated in this cohort, any potential benefit of prophylaxis should be interpreted as an inference from external evidence rather than as a direct finding of this study. Prospective multicenter studies incorporating immune reconstitution analyses are warranted.

## 1. Introduction

Although allo-HSCT offers a potentially curative treatment for patients with high-risk hematologic malignancies, opportunistic infections and GVHD remain the leading causes of non-relapse mortality (NRM) after transplantation. Among infectious complications, CMV reactivation continues to represent one of the most clinically significant causes of post-transplant morbidity and mortality despite major advances in antiviral prophylaxis and pre-emptive treatment strategies [1,2].

Profound post-transplant immunosuppression together with delayed immune reconstitution predisposes allo-HSCT recipients to viral reactivation, particularly CMV infection. CMV reactivation encompasses a broad clinical spectrum ranging from asymptomatic DNAemia to life-threatening end-organ disease and has been associated with increased overall mortality, non-relapse mortality, invasive fungal infections, renal dysfunction, poor graft function, prolonged hospitalization, and increased healthcare utilization [1].

The relationship between CMV reactivation and GVHD is complex and appears to be bidirectional. Severe GVHD and the immunosuppressive therapies required for its treatment clearly increase susceptibility to CMV reactivation. Conversely, whether CMV replication itself contributes to the development of GVHD remains controversial. Although several observational studies have reported an increased incidence of acute GVHD following CMV reactivation, pooled analyses and systematic reviews have produced inconsistent results, suggesting that the temporal interaction between these complications has not yet been fully clarified [3].

Interestingly, accumulating evidence indicates that CMV reactivation may also exert a graft-versus-leukemia (GVL) effect, particularly in patients with acute myeloid leukemia (AML). Several studies have demonstrated lower relapse rates among AML patients who developed CMV reactivation after allo-HSCT [4,5]. Recent evidence has suggested that a comparable reduction in relapse risk following CMV reactivation may also occur in patients with acute lymphoblastic leukemia (ALL). Nevertheless, further studies are needed to confirm these findings and clarify their biological basis and clinical implications [6]. Experimental and clinical studies suggest that this potential antileukemic effect may be mediated through the expansion of CMV-specific cytotoxic T lymphocytes and adaptive natural killer (NK) cells with enhanced antitumor activity [7,8].

Although letermovir prophylaxis has become the preferred strategy for CMV prevention in contemporary international guidelines [9,10], routine letermovir prophylaxis remains unavailable or is not universally implemented in many transplantation centers, including those in Türkiye. Consequently, CMV prevention in these centers continues to rely on herpes simplex virus (HSV) and varicella-zoster virus (VZV) prophylaxis with acyclovir, regular CMV surveillance, and pre-emptive antiviral therapy. This clinical setting provides a valuable opportunity to evaluate the natural history, temporal dynamics, and clinical consequences of CMV reactivation in the absence of universal letermovir prophylaxis.

Despite extensive investigation, several important questions remain unanswered. Most previous studies have evaluated CMV reactivation and GVHD as separate post-transplant complications without adequately accounting for the chronological sequence of these events. Furthermore, real-world data regarding long-term outcomes in patients managed exclusively with a pre-emptive CMV strategy remain limited. Therefore, the present study aimed to evaluate the incidence, risk factors, bidirectional temporal relationship, and prognostic significance of CMV reactivation and GVHD in adult allo-HSCT recipients managed without routine letermovir prophylaxis. To better characterize these temporal associations and minimize immortal-time bias, time-dependent Cox regression analyses were performed.

## 2. Materials and Methods

### 2.1. Study Population

This retrospective single-center cohort study included 100 adult patients (18–65 years) who underwent allogeneic hematopoietic stem cell transplantation (allo-HSCT) for hematologic malignancies at the Adult Hematology Stem Cell Transplantation Unit of Ankara Bilkent City Hospital between January 2016 and February 2025. The annual allo-HSCT volume at our center was not constant throughout the study period; the unit relocated in 2019 to a new facility with expanded capacity, accounting for the greater contribution of patients transplanted during the most recent five years.

The study protocol was approved by the Ethics Committee of Ankara Bilkent City Hospital (Approval No. 1-24-447; 7 August 2024), and all procedures were conducted in accordance with the Declaration of Helsinki. The approved protocol and data-collection period covered all transplantations performed throughout the ethics committee–approved study timeframe.

To minimize immortal-time bias and establish a homogeneous cohort for evaluating the temporal relationship between CMV reactivation and graft-versus-host disease (GVHD), a landmark design was applied. Patients who relapsed or died within the first 100 days after transplantation were excluded from the study.

The final day-100 landmark cohort comprised 100 patients who were alive and relapse-free at day + 100 and had complete required clinical and laboratory data. Patients with incomplete records were not included.

All transplant recipients and donors were CMV IgG seropositive before transplantation. Given the high CMV seroprevalence in Türkiye, the study cohort consisted exclusively of CMV-seropositive donor/recipient pairs (D+/R+), representing a population at high risk for post-transplant CMV reactivation.

### 2.2. Transplantation Protocol

Transplantations were performed using HLA-matched related donors, matched unrelated donors, haploidentical donors, and mismatched donors.

Patients with acute myeloid leukemia (AML), acute lymphoblastic leukemia (ALL), myelodysplastic syndrome (MDS), Hodgkin lymphoma, and other hematologic malignancies undergoing allo-HSCT were included.

Conditioning regimens were selected according to disease type, disease status, patient age, performance status, and comorbidity burden. Myeloablative conditioning (MAC) was administered to medically fit patients, whereas reduced-intensity conditioning (RIC) was preferred for patients considered unsuitable for MAC. Total body irradiation (TBI)-containing conditioning regimens were used in selected patients with ALL.

GVHD prophylaxis consisted of a post-transplant cyclophosphamide (PTCy)-based strategy followed by cyclosporine and mycophenolate mofetil in haploidentical and mismatched transplantations. Patients undergoing matched related or matched unrelated donor transplantation received cyclosporine combined with short-course methotrexate. Anti-thymocyte globulin (ATG) was administered in selected unrelated donor transplantations according to institutional practice.

### 2.3. CMV Monitoring and Pre-Emptive Therapy

All patients received oral acyclovir (400 mg three times daily) for herpes simplex virus (HSV) and varicella-zoster virus (VZV) prophylaxis from transplantation until at least day + 100.

Quantitative plasma CMV-DNA PCR was performed using the artus CMV QS-RGQ Kit (QIAGEN GmbH, Hilden, Germany) weekly during the first 100 days after transplantation. Monitoring was continued beyond day + 100 in patients considered at high risk for late CMV reactivation, defined as those with CMV reactivation before day + 100, active acute or chronic GVHD, or ongoing systemic corticosteroid or intensive immunosuppressive therapy. At day + 100, 73 patients met these criteria. Of these, 68 had experienced CMV reactivation before day + 100; this group included both patients with and without concurrent GVHD. The remaining five patients had developed GVHD before day + 100 without prior CMV reactivation. CMV-DNA PCR monitoring was continued beyond day + 100 in all 73 high-risk patients. In patients who developed GVHD after day + 100, monitoring was initiated or resumed and continued throughout immunosuppressive treatment and after its discontinuation until at least three consecutive weekly negative CMV-DNA PCR results had been documented.

Pre-emptive antiviral therapy was initiated when CMV-DNA reached ≥ 1000 IU/mL or demonstrated a ≥3-fold increase in viral load on two consecutive measurements, irrespective of clinical symptoms.

Viral load was monitored and reported in IU/mL because this was the unit generated by the institutional assay during the study period. Virological treatment failure was defined as persistent or increasing CMV-DNA levels despite at least 14 days of appropriately dosed antiviral therapy.

Intravenous ganciclovir or oral valganciclovir was selected according to clinical status. Patients with symptomatic CMV infection or requiring intravenous therapy were hospitalized and initially received intravenous ganciclovir. Once their symptoms improved and outpatient follow-up was considered feasible, they were discharged with oral valganciclovir to complete treatment. Foscarnet was not used as first-line therapy; two patients were switched from ganciclovir/valganciclovir to foscarnet due to ganciclovir-refractory disease.

The diagnosis and management of CMV end-organ disease were based on current international CMV consensus guidelines. Organ involvement was diagnosed according to compatible clinical manifestations together with radiologic, endoscopic, and/or histopathologic findings, supported whenever feasible by immunohistochemical or molecular detection of CMV in the affected tissue.

To align with recent international trials, clinically significant CMV infection (CS-CMVi) was defined as CMV end-organ disease or CMV DNAemia leading to initiation of pre-emptive antiviral treatment.

Antiviral therapy was administered for a minimum of 14 days and was discontinued after clinical improvement together with at least one documented undetectable plasma CMV-DNA PCR result. Treatment duration was extended when clinically indicated according to the patient’s clinical course and the extent of organ involvement.

The first CMV episode was defined as the first post-transplant occurrence of CMV DNAemia meeting the institutional threshold for initiation of pre-emptive antiviral therapy. A subsequent (recurrent) episode was defined as new CMV DNAemia requiring pre-emptive therapy after successful completion of treatment for the preceding episode and documentation of at least one undetectable plasma CMV-DNA PCR result. Persistent or increasing DNAemia during the preceding course of antiviral therapy was not considered a new episode but was classified as persistent DNAemia or virological treatment failure.

### 2.4. Diagnosis and Treatment of GVHD

Acute and chronic GVHD were diagnosed according to the European Society for Blood and Marrow Transplantation (EBMT) and National Institutes of Health (NIH) consensus criteria based on clinical manifestations and organ involvement.

Systemic corticosteroids were used as first-line therapy for acute GVHD. Methylprednisolone was initiated at a dose of 0.5–1 mg/kg/day in patients with isolated upper gastrointestinal involvement and at 1–2 mg/kg/day in those with lower gastrointestinal and/or hepatic involvement. Topical corticosteroids were added for patients with isolated cutaneous disease. Corticosteroids were gradually tapered after clinical response.

Steroid-refractory or steroid-intolerant patients received second-line therapies including ruxolitinib, ibrutinib, alpha-1 antitrypsin, and extracorporeal photopheresis according to clinical indication.

Chronic GVHD was classified as mild, moderate, or severe according to the NIH consensus criteria. Initial treatment consisted of systemic corticosteroids (methylprednisolone or equivalent at 0.5–1 mg/kg/day) while maintaining calcineurin inhibitor therapy. Organ-specific supportive therapies, including topical corticosteroids, mucosal agents, ocular lubricants, and other supportive measures, were administered when indicated. Patients with pulmonary GVHD received the FAM protocol (fluticasone, azithromycin, and montelukast). Steroid-refractory or steroid-intolerant patients were treated with ruxolitinib, ibrutinib, extracorporeal photopheresis, or other immunomodulatory therapies according to institutional practice.

### 2.5. Study Outcomes

The primary objective of the study was to evaluate the bidirectional temporal relationship between CMV reactivation and GVHD following allo-HSCT.

Secondary outcomes included CMV end-organ disease, platelet engraftment failure, overall survival (OS), relapse-free survival (RFS), and identification of clinical risk factors associated with CMV reactivation and GVHD.

Overall survival was defined as the time from transplantation to death from any cause or last follow-up. Relapse-free survival was defined as the time from transplantation to disease relapse, death, or last follow-up, whichever occurred first.

In the day-100 landmark survival analyses, time at risk began on day + 100. For clinical clarity, time in the Kaplan–Meier figures is displayed in months from transplantation; therefore, the first approximately 3.3 months contain no events by design. Patients who did not experience the event of interest (death or relapse) were censored at the date of their last clinical contact. Follow-up was complete for the entire landmark cohort up to the censoring date. The median follow-up was 34.8 months (95% CI, 28.4–41.1), with individual follow-up durations ranging from 3.1 to 126.6 months.

### 2.6. Statistical Analysis

Statistical analyses were performed using IBM SPSS Statistics for Windows, Version 27.0 (IBM Corp., Armonk, NY, USA).

The distribution of continuous variables was assessed using visual inspection and analytical tests for normality. Continuous variables are presented as mean ± standard deviation (SD) for normally distributed data or median with interquartile range (IQR) for non-normally distributed data. Categorical variables are expressed as frequencies and percentages.

Continuous variables were compared using the independent-samples *t*-test or the Mann–Whitney U test, as appropriate. Categorical variables were compared using the Pearson chi-square test or Fisher’s exact test.

Overall survival (OS) and relapse-free survival (RFS) were estimated using the Kaplan–Meier method and compared using the log-rank test. Survival estimates are reported with 95% confidence intervals (CIs).

To evaluate the chronological association between CMV reactivation and GVHD while minimizing immortal-time bias, bidirectional time-dependent Cox proportional hazards regression analyses were performed. CMV reactivation and GVHD were modeled separately as time-dependent covariates to assess their reciprocal effects on the subsequent occurrence of the other event. Hazard ratios (HRs) with 95% confidence intervals (CIs) were calculated.

All statistical tests were two-sided, and a *p*-value < 0.05 was considered statistically significant.

## 3. Results

### 3.1. Baseline Patient and Transplant Characteristics

A total of 100 adult patients who underwent allo-HSCT were included in the study. The baseline demographic, disease, and transplant characteristics of the study cohort are summarized in Table 1. The mean patient age was 40.4 ± 13.2 years, and 56 patients were male. Acute myeloid leukemia (AML) (58%) and acute lymphoblastic leukemia (ALL) (30%) were the most frequent indications for transplantation. Most patients (89%) were in first complete remission (CR1) at the time of transplantation. Stem cell grafts were predominantly obtained from matched sibling donors (50%) and mismatched unrelated donors (34%). Myeloablative conditioning (MAC) was administered in 64% of patients. For GVHD prophylaxis, post-transplant cyclophosphamide (PTCy) was used in 58% of patients, whereas cyclosporine plus mycophenolate mofetil was the most frequently administered immunosuppressive regimen (76%).

The median follow-up duration was 34.8 months (95% CI, 28.4–41.1).

### 3.2. Incidence and Temporal Relationship of CMV Reactivation and GVHD

During follow-up, CMV reactivation occurred in 72 patients (72%), whereas GVHD of any grade developed in 51 patients (51%). Among patients with GVHD, 32 (62.7%) developed acute GVHD and 19 (37.3%) developed chronic GVHD according to the conventional 100-day classification.

**Figure 1:**
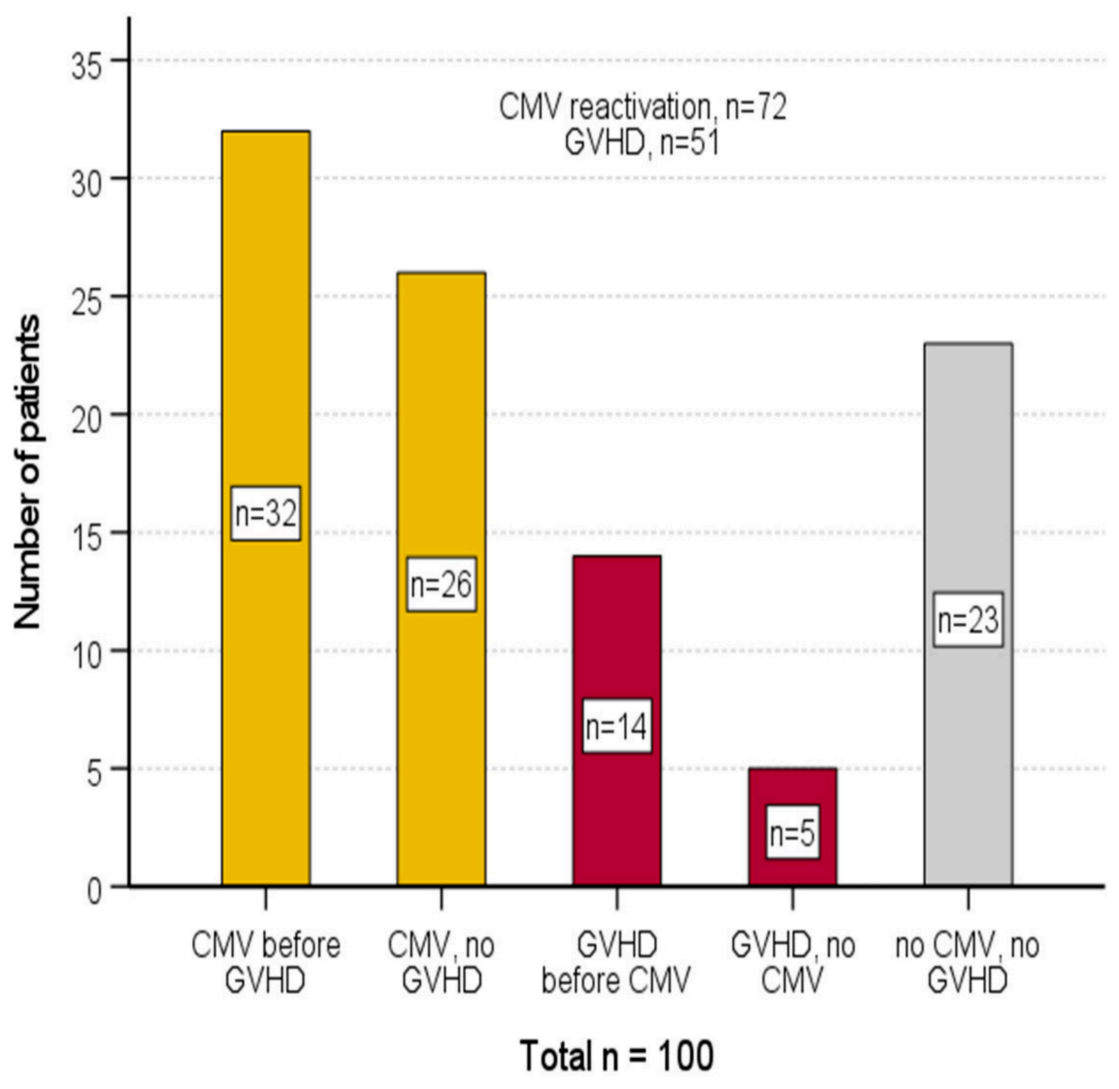
Chronological relationship between cytomegalovirus (CMV) reactivation and graft-versus-host disease (GVHD) in the study cohort (*n* = 100). The diagram illustrates the distribution of patients according to the occurrence and temporal sequence of CMV reactivation and GVHD following allogeneic hematopoietic stem cell transplantation.

Among the 46 patients who experienced both complications, CMV reactivation preceded the onset of GVHD in 32 patients (69.6%), whereas GVHD preceded CMV reactivation in 14 patients (30.4%). In addition, 26 patients experienced CMV reactivation without subsequent GVHD, while 5 patients developed GVHD without documented CMV reactivation. Twenty-three patients remained free of both CMV reactivation and GVHD throughout follow-up (Figure 1).

### 3.3. Factors Associated with CMV Reactivation and GVHD

Baseline demographic and transplant characteristics were compared according to CMV reactivation and GVHD status (Table 1).

**Figure 2:**
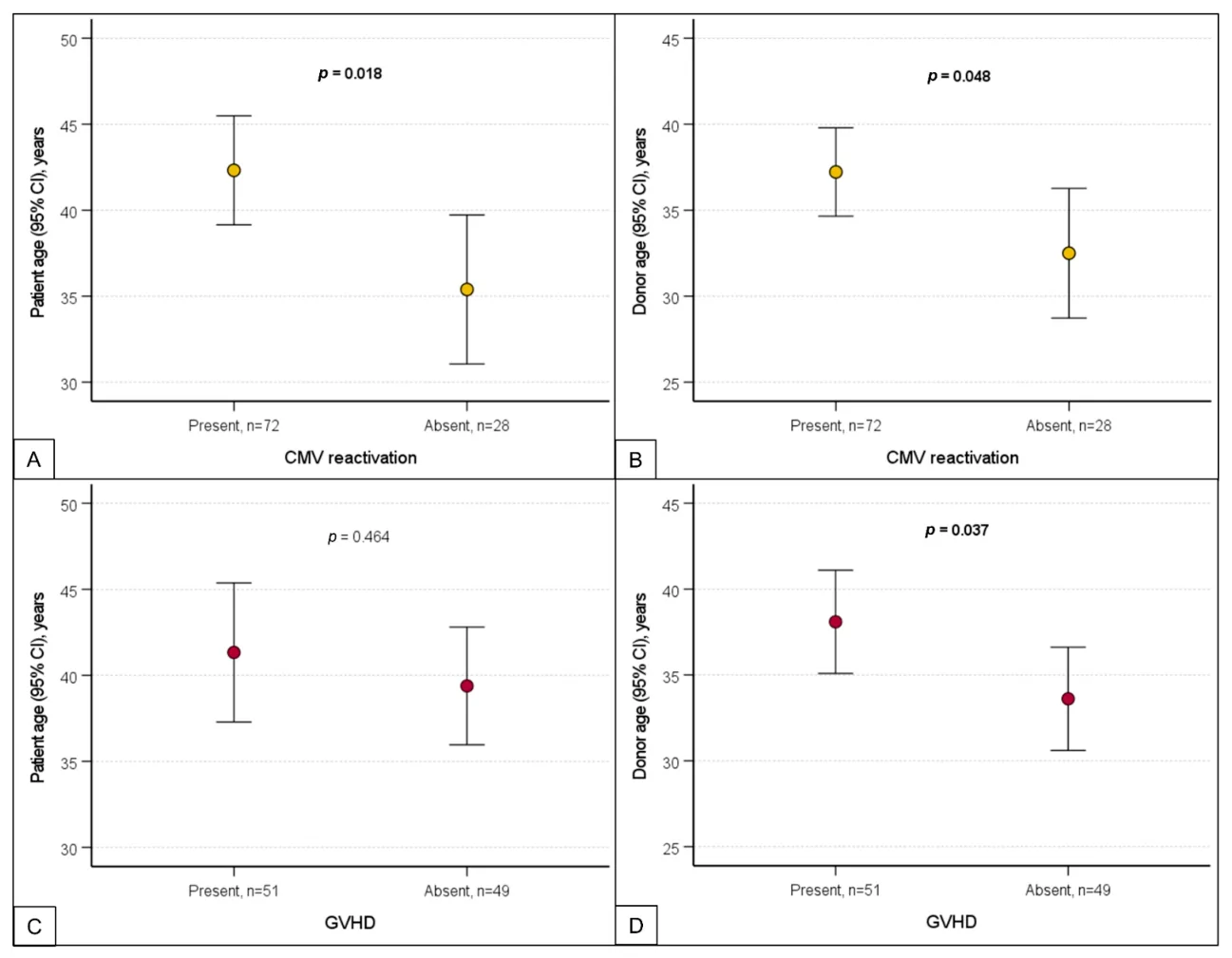
Comparison of recipient and donor ages according to post-transplant complications. (**A**) Recipient age according to CMV reactivation status. (**B**) Donor age according to CMV reactivation status. (**C**) Recipient age according to GVHD status. (**D**) Donor age according to GVHD status. Data are presented as mean values with 95% confidence intervals.

Patients who developed CMV reactivation were significantly older than those who did not (42.3 ± 13.5 vs. 35.4 ± 11.2 years, *p* = 0.018). Similarly, donor age was significantly higher among patients who experienced CMV reactivation (37.2 ± 10.9 vs. 32.5 ± 9.7 years, *p* = 0.048) (Figure 2).

Regarding GVHD, older donor age was the only baseline characteristic significantly associated with its development (38.1 ± 10.7 vs. 33.6 ± 10.5 years, *p* = 0.037) (Figure 2). No significant associations were observed between CMV reactivation or GVHD and donor type, conditioning intensity, measurable residual disease status before transplantation, in vivo T-cell depletion, donor–recipient sex matching, or ABO compatibility (all *p* > 0.05).

### 3.4. Clinical Characteristics of CMV Reactivation

The clinical characteristics of patients with CMV reactivation are summarized in Table 2.

The median time to first CMV reactivation was 37 days (IQR, 29–53), and the median duration of antiviral therapy for the first CMV episode was 29 days (IQR, 22–38). A single episode occurred in 46 patients (63.9%), two episodes in 21 patients (29.2%), three episodes in four patients (5.6%), and four episodes in one patient (1.4%). Among the 26 patients with recurrent CMV DNAemia, the second episode occurred at a median of 150 days (IQR, 90–210) after transplantation, whereas the third episode occurred at a median of 180 days. In the single patient who experienced four episodes, the fourth episode occurred on day 240 after transplantation.

All 72 patients with CMV reactivation received intravenous ganciclovir and/or oral valganciclovir as initial pre-emptive therapy. Two patients had persistent or increasing CMV-DNA despite at least 14 days of appropriately dosed therapy and were switched to foscarnet because of suspected ganciclovir resistance. No treatment modification due to toxicity was documented; however, drug-specific exposure durations and antiviral toxicity outcomes were not systematically captured and could not be reliably quantified.

CMV end-organ disease developed in 10 patients (13.9%), including CMV colitis (*n* = 7), pneumonitis (*n* = 1), retinitis (*n* = 1), and concurrent colitis with retinitis (*n* = 1).

Following CMV reactivation, platelet engraftment failure occurred in 25 patients (34.7%). Among these patients, 21 required eltrombopag because of persistent transfusion-dependent thrombocytopenia, whereas four experienced platelet recovery after viral clearance and discontinuation of antiviral therapy. Because cumulative exposure to individual antiviral agents and other time-varying causes of thrombocytopenia were not analyzed, this temporal observation should not be interpreted as evidence of a causal antiviral effect.

In bidirectional time-dependent Cox regression analyses, CMV reactivation was associated with an increased risk of subsequent GVHD (HR 2.93, 95% CI 1.51–5.69; *p* = 0.001). Conversely, GVHD was associated with an increased risk of subsequent CMV reactivation (HR 1.98, 95% CI 1.06–3.71; *p* = 0.032).

### 3.5. Characteristics of GVHD Episodes

The characteristics of sequential GVHD episodes are summarized in Table 3.

The first GVHD episode occurred in 51 patients at a median of 64 days (IQR, 31–150) after transplantation. Grade II GVHD was the most frequent presentation (66.7%). The skin (54.9%) and gastrointestinal tract (23.5%) were the organs most commonly involved during the initial episode.

A second GVHD episode developed in 27 patients at a median of 150 days (IQR, 57–240). Grade II disease remained the predominant severity (66.7%), whereas gastrointestinal involvement (37.0%) was the most common manifestation, followed by skin (18.5%) and liver involvement (18.5%).

A third GVHD episode occurred in 11 patients at a median of 215 days (IQR, 150–360). Liver (36.4%) and pulmonary involvement (27.3%) predominated during these episodes, whereas skin and gastrointestinal involvement were each observed in 9.1% of patients.

### 3.6. Survival Outcomes

After a median follow-up of 34.8 months (95% CI, 28.4–41.1), disease relapse occurred in 19 patients (19%), and 30 patients (30%) died. Among these deaths, 16 (53.3%) were attributable to non-relapse mortality.

The estimated 1-year and 2-year relapse-free survival (RFS) rates were 81.7% (95% CI, 73.9–89.5) and 70.2% (95% CI, 60.4–80.0), respectively. The corresponding overall survival (OS) rates were 89.3% (95% CI, 83.0–95.6) and 75.5% (95% CI, 66.3–84.7), respectively. Median OS and RFS were not reached during follow-up.

**Figure 3:**
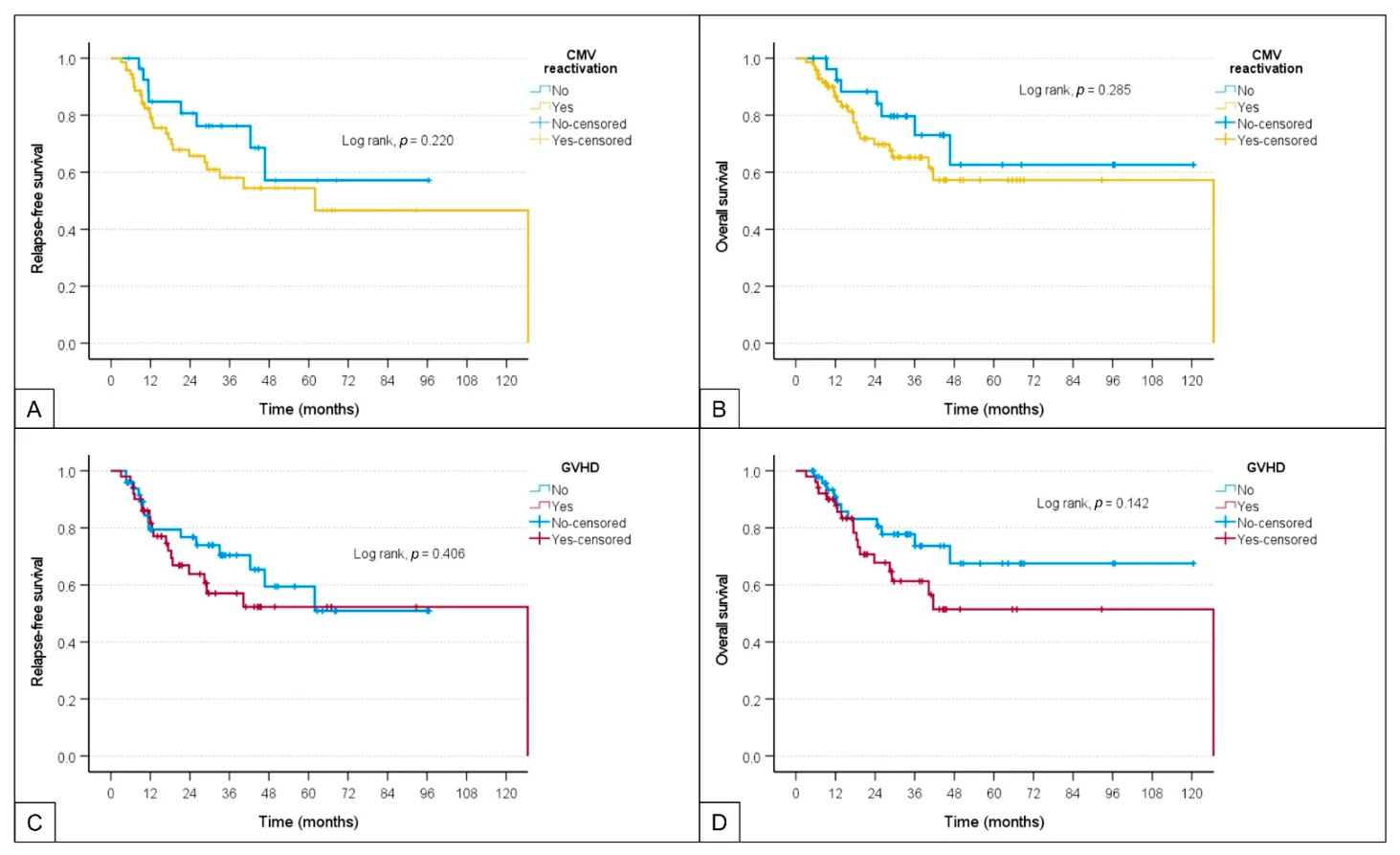
Kaplan–Meier survival analyses of the entire study cohort (*n* = 100). (**A**) Relapse-free survival (RFS) according to CMV reactivation status. (**B**) Overall survival (OS) according to CMV reactivation status. (**C**) RFS according to GVHD status. (**D**) OS according to GVHD status. Survival curves were compared using the log-rank test. Note: Due to the day-100 landmark design, there are no events in the first approximately 3.3 months (100 days) of follow-up.

Kaplan–Meier analyses demonstrated no significant differences in RFS (log-rank *p* = 0.220; Figure 3A) or OS (log-rank *p* = 0.285; Figure 3B) according to CMV reactivation status.

Similarly, neither RFS (log-rank *p* = 0.406; Figure 3C) nor OS (log-rank *p* = 0.142; Figure 3D) differed significantly between patients who developed GVHD and those who did not.

### 3.7. AML Subgroup Analysis

Because AML represented the largest disease subgroup, a predefined subgroup analysis was performed in 58 patients.

According to disease-risk stratification, 42 patients (72.4%) were classified as intermediate-risk and 16 (27.6%) as high-risk AML. The incidence of CMV reactivation did not differ significantly between the intermediate- and high-risk groups (76.2% vs. 56.3%, *p* = 0.197). Likewise, AML risk category was not significantly associated with GVHD development (57.1% vs. 31.3%, *p* = 0.078).

**Figure 4:**
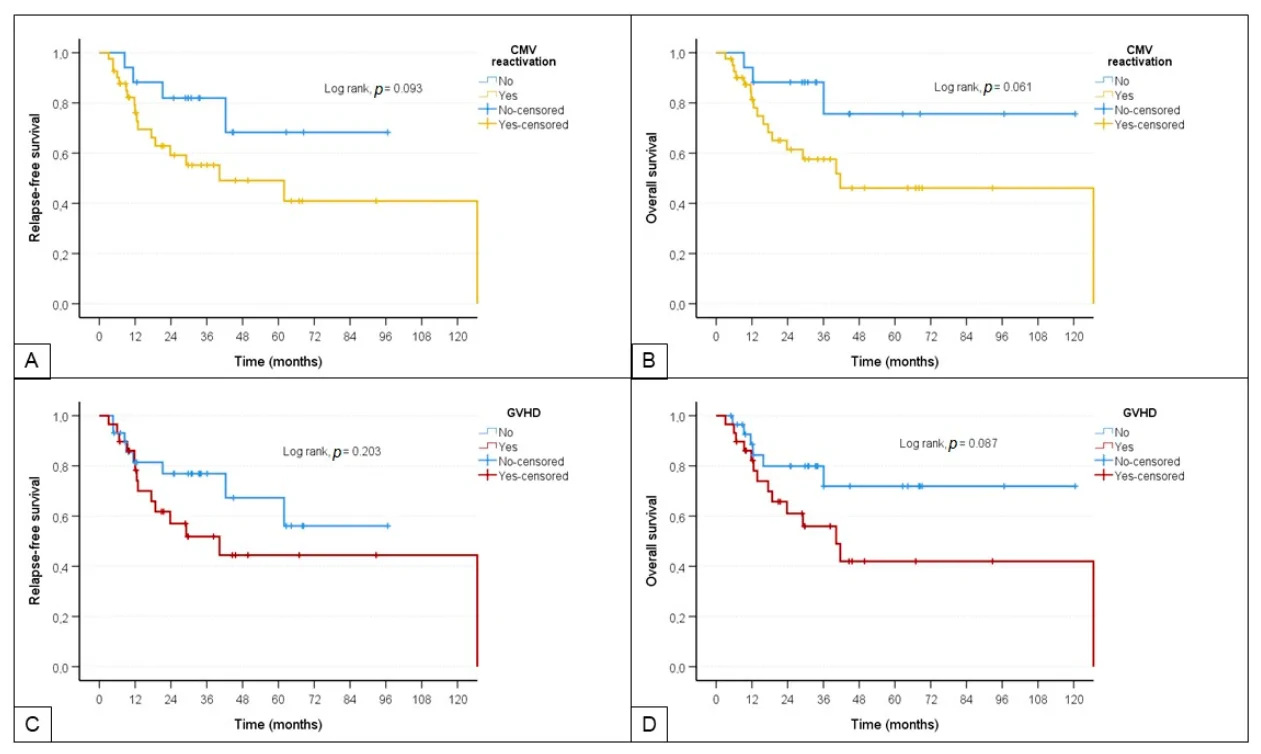
Kaplan–Meier survival analyses of the acute myeloid leukemia (AML) subgroup (*n* = 58). (**A**) Relapse-free survival (RFS) according to CMV reactivation status. (**B**) Overall survival (OS) according to CMV reactivation status. (**C**) RFS according to GVHD status. (**D**) OS according to GVHD status. Survival curves were compared using the log-rank test. Note: Due to the day-100 landmark design, there are no events in the first approximately 3.3 months (100 days) of follow-up.

Kaplan–Meier analyses performed within the AML subgroup showed a trend toward inferior RFS (log-rank *p* = 0.093; Figure 4A) and OS (log-rank *p* = 0.061; Figure 4B) among patients who experienced CMV reactivation compared with those who did not.

Similarly, AML patients who developed GVHD showed a trend toward inferior OS (log-rank *p* = 0.087; Figure 4D), whereas no significant difference was observed in RFS (log-rank *p* = 0.203; Figure 4C).

## 4. Discussion

CMV remains one of the most clinically significant infectious complications following allo-HSCT despite substantial advances in antiviral prophylaxis, molecular diagnostics, and supportive care [1,2,8,11]. The introduction of letermovir has fundamentally changed CMV prevention and is currently recommended as the standard strategy for primary prophylaxis in CMV-seropositive allo-HSCT recipients according to contemporary international guidelines [9,10]. Nevertheless, many transplantation centers, particularly those with limited access to letermovir, continue to rely on intensive virological surveillance coupled with a pre-emptive treatment strategy. Therefore, evaluating the effectiveness and limitations of standardized pre-emptive CMV management remains highly relevant in current clinical practice. In this real-world cohort of CMV-seropositive donor–recipient pairs managed without routine letermovir prophylaxis, long-term survival did not significantly differ according to CMV status; nevertheless, clinically meaningful CMV-related morbidity remained frequent under pre-emptive management.

CMV reactivation occurred in 72% of patients, whereas GVHD developed in 51%, highlighting the substantial burden of post-transplant infectious and immune-mediated complications. The median time to CMV reactivation was 37 days, consistent with previous reports demonstrating that CMV replication predominantly occurs during the period of profound adaptive immune deficiency early after transplantation [2,8,12].

One of the most important findings of our study was the temporal sequence between CMV reactivation and GVHD. CMV reactivation preceded GVHD in the majority of patients who experienced both complications, supporting an increasingly recognized bidirectional interaction between viral replication and alloimmune activation. Although the retrospective design precludes definitive causal inference, this chronological sequence is biologically plausible and suggests that CMV reactivation and GVHD represent interconnected manifestations of dysregulated immune recovery rather than independent post-transplant events [3,7,11].

Importantly, this observation was supported by bidirectional time-dependent Cox regression analyses. Unlike conventional survival analyses, time-dependent Cox modeling incorporates the exact timing of each event during follow-up and minimizes immortal-time bias. CMV reactivation nearly tripled the subsequent hazard of GVHD, whereas GVHD approximately doubled the hazard of subsequent CMV reactivation, providing statistical evidence for a dynamic reciprocal association between these complications.

Several biological mechanisms may explain this interaction. CMV replication induces endothelial activation, enhances antigen presentation, increases the production of pro-inflammatory cytokines including interleukin-6 and tumor necrosis factor-α, and promotes expansion of donor-derived effector T lymphocytes [11,13]. These processes amplify alloreactivity and may facilitate the initiation or progression of GVHD. Conversely, GVHD itself together with the immunosuppressive therapy required for its treatment impairs CMV-specific cellular immunity, thereby facilitating viral reactivation and establishing a self-perpetuating cycle of inflammation and immune dysfunction [3,11,14,15].

Our findings are consistent with previous studies reporting a close chronological association between CMV replication and acute GVHD. More recent evidence further suggests that impaired CMV-specific immune reconstitution, persistent inflammatory signaling, endothelial dysfunction, and delayed recovery of virus-specific T-cell immunity are key mechanisms linking CMV reactivation and clinically significant GVHD [3,9,11,13]. Accordingly, current international recommendations increasingly emphasize immune monitoring in addition to quantitative CMV-DNA surveillance because viral load alone does not fully reflect the host’s capacity to control CMV replication [9,10].

Another noteworthy finding was the identification of older donor age as a shared risk factor for both CMV reactivation and GVHD. Although donor age has long been recognized as an important determinant of transplant outcomes, its relationship with CMV reactivation has received comparatively little attention. Age-related immunosenescence may reduce T-cell repertoire diversity, impair immune reconstitution, and promote a pro-inflammatory immune phenotype, thereby predisposing recipients to both viral reactivation and alloimmune complications [16,17].

Despite rigorous weekly CMV-DNA surveillance and the prompt initiation of pre-emptive antiviral therapy, 13.9% of patients developed CMV end-organ disease, predominantly CMV colitis. This incidence is higher than that reported in centers implementing optimized pre-emptive strategies and likely reflects the cumulative immunosuppressive burden associated with GVHD, prolonged corticosteroid exposure, and delayed immune reconstitution. Two patients met the prespecified clinical definition of virological treatment failure and were switched to foscarnet because of suspected ganciclovir resistance. Because genotypic resistance testing was not routinely performed, UL97 or UL54 mutations could not be confirmed, and resistance remains a clinical rather than molecular diagnosis in these cases [9,18,19].

Another clinically important finding was the high incidence of platelet engraftment failure following CMV reactivation, affecting 25 patients (34.7%). Among these, 21 required salvage therapy with eltrombopag because of persistent transfusion-dependent thrombocytopenia, whereas four achieved platelet recovery after viral clearance and discontinuation of antiviral therapy. The association between CMV reactivation and impaired platelet recovery is likely multifactorial and may reflect direct viral marrow suppression, the known myelosuppressive potential of ganciclovir/valganciclovir, underlying marrow dysfunction, concomitant medications, GVHD, and cumulative immunosuppression. However, because drug-specific cumulative exposure was not analyzed, the independent contribution of antiviral myelotoxicity cannot be determined from this study [11,18,20]. Recent studies have demonstrated that eltrombopag represents an effective rescue strategy for refractory post-transplant thrombocytopenia without increasing GVHD risk [21,22].

It is important to acknowledge that distinguishing direct viral bone marrow suppression from antiviral myelotoxicity is challenging in a retrospective cohort. Foscarnet was used in only two patients after suspected ganciclovir resistance and was not selected as first-line therapy because of baseline cytopenias; therefore, agent-specific hematologic toxicity could not be compared. Baseline marrow status, CMV severity, GVHD, and concomitant immunosuppression remain potential confounders. Maribavir, which lacks the myelosuppressive effects of (val)ganciclovir and the nephrotoxicity of foscarnet, is an important option for refractory or resistant CMV and may reduce treatment-related morbidity [23].

Collectively, these findings support an evolving perspective on CMV management. Whereas historical prevention strategies primarily focused on reducing CMV disease and mortality, contemporary evidence indicates that the principal burden of CMV lies in transplant-related morbidity, including delayed hematopoietic recovery, prolonged hospitalization, repeated antiviral exposure, increased healthcare utilization, and impaired quality of life [9,23,24]. Our findings support this concept by demonstrating that although survival outcomes did not significantly differ according to CMV reactivation status, pre-emptive management did not adequately prevent clinically meaningful CMV-related complications.

Interestingly, although previous studies have suggested a protective graft-versus-leukemia effect associated with CMV reactivation, particularly in AML, our subgroup analysis showed a trend toward inferior RFS and OS among AML patients with CMV reactivation. The high burden of CMV-related morbidity, the need for antiviral therapy, subsequent GVHD, and increased immunosuppressive burden may have increased non-relapse mortality and obscured a potential antileukemic benefit. The relatively small AML subgroup also limited statistical power. Moreover, the absence of a competing-risks analysis separating relapse from non-relapse mortality constrains the interpretation of RFS in the setting of substantial transplant-related toxicity. Thus, the net clinical effect of CMV reactivation in AML may depend on the balance between a potential GVL effect and CMV- and treatment-related morbidity.

This study has several limitations. Its retrospective single-center design limits the ability to establish causality despite time-dependent Cox regression. The final sample size was limited by the strict inclusion of D+/R+ pairs, the day-100 landmark design, and the exclusion of patients with incomplete records; consequently, statistical power was limited. No formal pre-study sample-size or power calculation was performed. The landmark design also excluded patients with early death or relapse; therefore, the survival findings apply only to patients who were alive and relapse-free at day + 100 and cannot be generalized to the entire allo-HSCT population. Reliable cumulative corticosteroid dose and duration data were not available for the entire cohort; consequently, corticosteroid exposure could not be evaluated as a time-dependent covariate. Detailed CMV viral-load kinetics, including peak viral load, duration of DNAemia, and area under the curve, could not be systematically analyzed. Antiviral treatment duration was available only for the first CMV episode; cumulative antiviral exposure across all episodes, drug-specific treatment days, and switches between intravenous ganciclovir and oral valganciclovir were not systematically recorded. Antiviral toxicity outcomes, including new-onset neutropenia, acute kidney injury, electrolyte disturbances, and treatment modification or discontinuation because of toxicity, could not be reliably quantified. Consequently, the independent contribution of cumulative antiviral exposure to platelet recovery could not be evaluated. CMV-specific immune reconstitution, routine genotypic resistance testing, and immune monitoring were also unavailable, precluding mechanistic evaluation of host antiviral immunity.

Nevertheless, the study also has important strengths. All patients underwent standardized weekly CMV monitoring using a uniform pre-emptive treatment protocol, all donor–recipient pairs were CMV seropositive, and bidirectional time-dependent Cox regression analyses were performed to account for the exact chronological sequence of CMV reactivation and GVHD, thereby minimizing immortal-time bias. Furthermore, the study reflects real-world clinical practice in a transplantation center where routine letermovir prophylaxis is unavailable.

Importantly, because no patient in our cohort received letermovir, direct comparisons cannot be made. Nevertheless, when interpreted alongside external evidence, the high burden of CMV-associated morbidity observed in our cohort provides indirect support for current international recommendations favoring letermovir prophylaxis in CMV-seropositive allo-HSCT recipients. Randomized trials and real-world studies have shown that letermovir reduces clinically significant CMV infection, exposure to myelotoxic antiviral therapy, CMV end-organ disease, and CMV-related hospitalization [9,10,24,25].

## 5. Conclusions

CMV reactivation remains highly prevalent after allo-HSCT and frequently precedes the development of GVHD, consistent with a dynamic bidirectional association between these complications. Older donor age was associated with both CMV reactivation and GVHD. Although long-term overall and relapse-free survival did not significantly differ according to CMV status in this day-100 landmark cohort managed with a standardized pre-emptive strategy, clinically significant morbidity, including CMV end-organ disease and impaired platelet recovery, remained frequent. The relative contributions of CMV infection, antiviral exposure, baseline marrow dysfunction, GVHD, and concomitant immunosuppression to impaired platelet recovery could not be separated in this retrospective analysis. While letermovir was not evaluated in this study, our observations, considered together with external evidence, support prospective evaluation of prevention strategies using both survival and morbidity endpoints. Prospective multicenter studies incorporating drug-specific antiviral exposure and CMV-specific immune reconstitution monitoring are warranted.

## Figures and Tables

**Table 1:** Baseline and transplant characteristics according to CMV reactivation and GVHD status.

Characteristics	Total, *n* = 100	CMV Reactivation		*p* Value	GVHD		*p* Value
		Present, *n* = 72	Absent, *n* = 28		Present, *n* = 51	Absent, *n* = 49	
Age, mean ± SD, years	40.4 ± 13.2	42.3 ± 13.5	35.4 ± 11.2	0.018	41.3 ± 14.4	39.4 ± 11.9	0.464
Male sex, *n* (%)	56 (56.0)	44 (61.1)	12 (42.9)	0.099	29 (56.9)	27 (55.1)	0.859
Diagnosis, *n* (%) AML ALL MDS Hodgkin lymphoma CML/MPN	58 (58.0) 30 (30.0) 5 (5.0) 4 (4.0) 3 (3.0)	41 (56.9) 23 (31.9) 2 (2.8) 3 (4.2) 3 (4.2)	17 (60.7) 7 (25.0) 3 (10.7) 1 (3.6) 0 (0.0)	0.393	29 (56.9) 15 (29.4) 2 (3.9) 3 (5.9) 2 (3.9)	29 (59.2) 15 (30.6) 3 (6.1) 1 (2.0) 1 (2.0)	0.828
HCT-CI score, *n* (%) 0 1–2 ≥3	68 (68.0) 26 (26.0) 6 (6.0)	48 (66.7) 19 (26.4) 5 (6.9)	20 (71.4) 7 (25.0) 1 (3.6)	0.793	34 (66.7) 14 (27.5) 3 (5.9)	34 (69.4) 12 (24.5) 3 (6.1)	0.945
Disease status, *n* (%) CR1 CR2 NA	89 (89.0) 10 (10.0) 1 (1.0)	64 (88.9) 8 (11.1) 0 (0.0)	25 (89.3) 2 (7.1) 1 (3.6)	0.236	43 (84.3) 8 (15.7) 0 (0.0)	46 (93.9) 2 (4.1) 1 (2.0)	0.097
Pre-transplant MRD, *n* (%) Negative Positive Unknown/NA	36 (36.0) 49 (49.0) 15 (15.0)	26 (36.1) 34 (47.2) 12 (16.7)	10 (35.7) 15 (53.6) 3 (10.7)	0.725	18 (35.3) 23 (45.1) 10 (19.6)	18 (36.7) 26 (53.1) 5 (10.2)	0.404
Donor type, *n* (%) MSD MUD MMUD Haploidentical	50 (50.0) 11 (11.0) 34 (34.0) 5 (5.0)	32 (44.4) 11 (15.3) 25 (34.7) 4 (5.6)	18 (64.3) 0 (0.0) 9 (32.1) 1 (3.6)	0.109	25 (49.0) 7 (13.7) 18 (35.3) 1 (2.0)	25 (51.0) 4 (8.2) 16 (32.7) 4 (8.2)	0.441
Donor age, mean ± SD, years	35.9 ± 10.8	37.2 ± 10.9	32.5 ± 9.7	0.048	38.1 ± 10.7	33.6 ± 10.5	0.037
Donor–recipient sex match, *n* (%) Matched Mismatched	56 (56.0) 44 (44.0)	41 (56.9) 31 (43.1)	15 (53.6) 13 (46.4)	0.760	27 (52.9) 24 (47.1)	29 (59.2) 20 (40.8)	0.530
ABO compatibility, *n* (%) Matched Mismatched	48 (48.0) 52 (52.0)	32 (44.4) 40 (55.6)	16 (57.1) 12 (42.9)	0.254	22 (43.1) 29 (56.9)	26 (53.1) 23 (46.9)	0.321
Conditioning intensity, *n* (%) Myeloablative Reduced intensity	64 (64.0) 36 (36.0)	42 (58.3) 30 (41.7)	22 (78.6) 6 (21.4)	0.058	30 (58.8) 21 (41.2)	34 (69.4) 15 (30.6)	0.271
Anti-thymocyte globulin use, *n* (%)	23 (23.0)	15 (20.8)	8 (28.6)	0.409	12 (23.5)	11 (22.4)	0.898
Cyclophosphamide use, *n* (%)	58 (58.0)	44 (61.1)	14 (50.0)	0.312	26 (51.0)	32 (65.3)	0.147
GVHD prophylaxis, *n* (%) Cyclosporine + MMF Cyclosporine + MTX	76 (76.0) 24 (24.0)	58 (80.6) 14 (19.4)	18 (64.3) 10 (35.7)	0.087	38 (74.5) 13 (25.5)	38 (77.6) 11 (22.4)	0.722

Abbreviations: ALL, acute lymphoblastic leukemia; AML, acute myeloid leukemia; CML, chronic myeloid leukemia; CMV, cytomegalovirus; CR, complete remission; GVHD, graft-versus-host disease; HCT-CI, hematopoietic cell transplantation-specific comorbidity index; MDS, myelodysplastic syndrome; MMF, mycophenolate mofetil; MMUD, mismatched unrelated donor; MPN, myeloproliferative neoplasm; MSD, matched sibling donor; MTX, methotrexate; MUD, matched unrelated donor; MRD, minimal residual disease; NA, not applicable; SD, standard deviation; CR1, Complete remission 1; CR2, Complete remission 2.

**Table 2:** Clinical characteristics and outcomes of CMV reactivation (*n* = 72).

Parameters	Findings
Time to CMV reactivation, median (IQR), days	37 (29–53)
Duration of antiviral therapy for the first CMV episode, median (IQR), days	29 (22–38)
Number of CMV reactivation episodes, *n* (%) 1 2 3 4	46 (63.9) 21 (29.2) 4 (5.6) 1 (1.4)
Time to second CMV episode, median (IQR), days	150 (90–210)
Time to third CMV episode, median, days	180
Time to fourth CMV episode, days	240
CMV disease, *n* (%) Colitis Pneumonitis Retinitis Colitis + retinitis	10 (13.9) 7/10 1/10 1/10 1/10
Platelet engraftment failure after CMV reactivation, *n* (%)	25 (34.7)

Abbreviations: CMV, cytomegalovirus; IQR, interquartile range.

**Table 3:** Clinical characteristics, severity, and target organ involvement across sequential GVHD episodes.

Parameters	1st GVHD	2nd GVHD	3rd GVHD
Number of patients	51	27	11
Grade, *n* (%) I II III IV	9 (17.6) 34 (66.7) 6 (11.8) 2 (3.9)	4 (14.8) 18 (66.7) 3 (11.1) 2 (7.4)	2 (18.2) 6 (54.5) 2 (18.2) 1 (9.1)
Time to GVHD, median (IQR), days	64 (31–150)	150 (57–240)	215 (150–360)
Organ involved, *n* (%) Skin Gastrointestinal tract Liver Oral cavity Eyes Lungs	28 (54.9) 12 (23.5) 7 (13.7) - 4 (7.8) -	5 (18.5) 10 (37.0) 5 (18.5) 1 (3.7) 2 (7.4) 4 (14.8)	1 (9.1) 1 (9.1) 4 (36.4) - 2 (18.2) 3 (27.3)

Abbreviations: GVHD, graft-versus-host disease, IQR, interquartile range.

## Data Availability

The datasets analyzed during the current study are available from the corresponding author upon reasonable request. The data are not publicly available due to ethical restrictions and the need to protect patient confidentiality.

## Abbreviations

ALL	Acute lymphoblastic leukemia
allo-HSCT	Allogeneic hematopoietic stem cell transplantation
AML	Acute myeloid leukemia
ATG	Anti-thymocyte globulin
CI	Confidence interval
CML	Chronic myeloid leukemia
CMV	Cytomegalovirus
CR	Complete remission
CR1	Complete remission 1
CR2	Complete remission 2
DNA	Deoxyribonucleic acid
EBMT	European Society for Blood and Marrow Transplantation
ECIL	European Conference on Infections in Leukaemia
FAM	Fluticasone, azithromycin, and montelukast
GenAI	Generative artificial intelligence
GVHD	Graft-versus-host disease
GVL	Graft-versus-leukemia
HCT-CI	Hematopoietic cell transplantation-specific comorbidity index
HLA	Human leukocyte antigen
HR	Hazard ratio
HSV	Herpes simplex virus
IgG	Immunoglobulin G
IQR	Interquartile range
MAC	Myeloablative conditioning
MDS	Myelodysplastic syndrome
MMF	Mycophenolate mofetil
MMUD	Mismatched unrelated donor
MPN	Myeloproliferative neoplasm
MRD	Minimal residual disease
MSD	Matched sibling donor
MTX	Methotrexate
MUD	Matched unrelated donor
NA	Not applicable
NIH	National Institutes of Health
NK	Natural killer
NRM	Non-relapse mortality
OS	Overall survival
PCR	Polymerase chain reaction
PET/CT	Positron emission tomography/computed tomography
PTCy	Post-transplant cyclophosphamide
RFS	Relapse-free survival
RIC	Reduced-intensity conditioning
SD	Standard deviation
TBI	Total body irradiation
VZV	Varicella-zoster virus
